# A glycoside analog of mammalian oligomannose formulated with a TLR4-stimulating adjuvant elicits HIV-1 cross-reactive antibodies

**DOI:** 10.1038/s41598-021-84116-w

**Published:** 2021-02-25

**Authors:** Jean-François Bruxelle, Tess Kirilenko, Nino Trattnig, Yiqiu Yang, Matteo Cattin, Paul Kosma, Ralph Pantophlet

**Affiliations:** 1 Faculty of Health Sciences, Simon Fraser University, Burnaby, BC Canada; 2 Department of Chemistry, University of Natural Resources and Life Sciences, Vienna, Austria; 3 Department of Molecular Biology and Biochemistry, Simon Fraser University, Burnaby, BC Canada; 4 Present Address: AbCellera Biologics Inc., Vancouver, BC Canada; 5 Present Address: Department of Chemical Biology and Drug Discovery, Utrecht University, Utrecht, The Netherlands

**Keywords:** Adaptive immunity, Vaccines, Immunology, Infectious diseases, Glycobiology

## Abstract

The occurrence of oligomannose-specific broadly neutralizing antibodies (bnAbs) has spurred efforts to develop immunogens that can elicit similar antibodies. Here, we report on the antigenicity and immunogenicity of a CRM_197_-conjugate of a previously reported oligomannose mimetic. Oligomannose-specific bnAbs that are less dependent on interactions with the HIV envelope protein sequence showed strong binding to the glycoconjugates, with affinities approximating those reported for their cognate epitope. The glycoconjugate is also recognized by inferred germline precursors of oligomannose-specific bnAbs, albeit with the expected low avidity, supporting its potential as an immunogen. Immunization of human-antibody transgenic mice revealed that only a TLR4-stimulating adjuvant formulation resulted in antibodies able to bind a panel of recombinant HIV trimers. These antibodies bound at relatively modest levels, possibly explaining their inability to neutralize HIV infectivity. Nevertheless, these findings contribute further to understanding conditions for eliciting HIV-cross-reactive oligomannose-specific antibodies and inform on next steps for improving on the elicited response.

## Introduction

An effective vaccine is still considered the best way to blunt the global spread of HIV/AIDS. However, the design of such a vaccine continues to face scientific challenges, including the development of a component that can elicit broadly reactive HIV-neutralizing antibodies (bnAbs). Extensive investigations have led to the delineation of seven antigenic sites on the HIV-1 envelope glycoprotein spike (Env) that are targets of bnAbs, among which is a relatively conserved patch of oligomannose-type glycans^1^. NAbs to this glycan patch are among the most potent and cross-reactive^2^—a recognition that has incentivized attempts to design immunogens capable of eliciting equivalent antibodies.

A common belief is that the elicitation of antibodies to the aforementioned glycan patch is likely to be hampered by B cell tolerance mechanisms, because of the ‘self’ nature of the targeted molecules. One potential way to overcome tolerogenic restrictions is to engineer glycosides that appear ‘foreign’ to the immune system but that sufficiently mimic oligomannose to yield cross-reactive antibodies. Not long ago, we began probing this idea based on the discovery of a bacterial oligosaccharide with resemblance to oligomannose^3,4^ and knowledge that mimicry of mammalian oligosaccharides by bacterial glycans can overcome natural tolerance. We previously reported on a neoglycoside that when conjugated to BSA elicits modest levels of nAbs against select HIV strains following immunization of human-antibody transgenic rats^5^ and are working on strategies to heighten elicitation of the desired antibodies, for example by introducing distinctive bacterial constituents^6^.

One of the caveats of our previous study was the use of BSA as a carrier protein. Although convenient, BSA is not a clinically acceptable carrier. To address this caveat, we have chosen CRM_197_, a non-toxic mutant of diphtheria toxin. CRM_197_ is now common in marketed glycoconjugate vaccines and is known to stimulate robust T-follicular helper cell (Tfh) responses^7–10^, a feature that is important for affinity maturation of anti-glycan Abs^11^. We are however not the first to use CRM_197_ for the design of glycoconjugates to target oligomannose on HIV; it has been used by others in the past in the design of glycoconjugates with dense glycan clusters in an attempt to elicit 2G12-like oligomannose-specific bnAbs^12^. We report here on the binding interaction of oligomannose-specific bnAbs from several different families along with their inferred germline (gl) precursors, i.e., the unmutated common ancestor, to a CRM_197_ conjugate of our lead glycoside^5^.

Another limitation of our previous study was the choice of adjuvant. Several adjuvants are now used in human vaccines^13^, albeit that only alum and MF59 are used in current glycoconjugate vaccines. MF59 is better than alum with some glycoconjugate vaccines^14^ and therefore an MF59-like formulation (Addavax; Invivogen) was used in our previous work. However, because MF59 does not work well with all glycoconjugates^15^, we wished to evaluate how different categories of clinically relevant adjuvant formulations might impact the immunogenicity of our glycoside conjugate. Specifically, we report here on the efficacy of adjuvants exemplifying three different categories—aluminum salt (Alhydrogel), squalene oil-in-water (AddaVax), TLR4 agonist (GLA-SE)^16–18^—when formulated with our glycoconjugate to induce HIV-cross-reactive antibody responses. Here, we used the Trianni mouse model, which express a full human antibody repertoire, to evaluate the immunogenicity of our conjugate in animals that evolve human-like antibody responses^19^. We show that only the immunization with the oligomannose mimetic conjugate formulated in GLA-SE elicited antibody responses with capacity to bind the oligomannose mimetic. Sera from these animals also bound various recombinant HIV SOSIP gp140s. However, analysis of this antibody response revealed that these two responses are distinct, with those specific for the mimetic being predominantly of the IgG3 subclass whereas those specific for HIV Env were largely IgG2. Although immunizing repeatedly with the same conjugate was not sufficient to yield meaningful nAbs, our findings provide clues for possible ways to augment the level of cross-reactivity to achieve virus neutralization.

## Results

### Members of the oligomannose-patch specific PGT128/130 bnAb family bind avidly to CRM_197_-conjugated glycomimetic NIT211, with affinities approximating those reported for recombinant HIV

**Figure 1:**
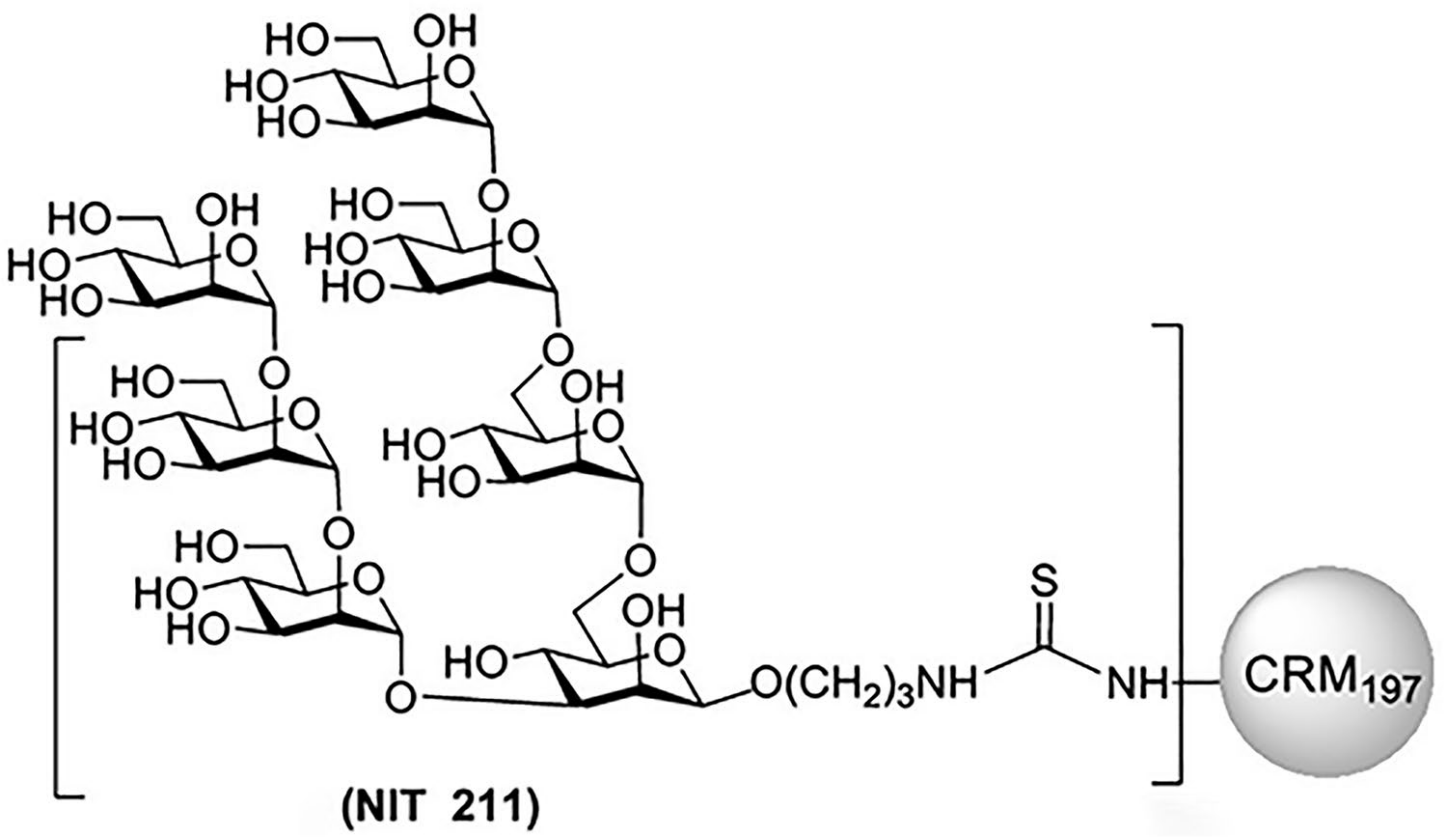
Schematic of neoglycoconjugate NIT211. Shown is the chemical structure of a synthetic oligomannose mimetic^5^ conjugated onto CRM_197_. The number of glycoside molecules per CRM_197_ for each conjugate was determined by MALDI-TOF^6^ and ranged from 2–6 glycosides. Image ^6^ adapted from Trattnig et al.

**Figure 2:**
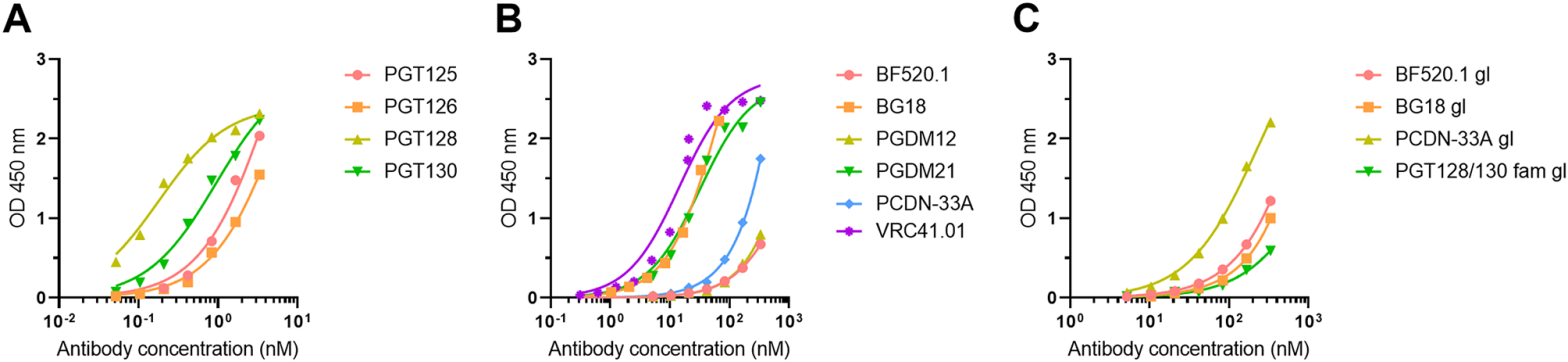
NIT211 conjugate is recognized by oligomannose-specific bnAbs and their germline precursors. NIT211 (4.1 glycosides per CRM_197_) was coated as solid-phase antigen onto ELISA plate wells at 5 µg/ml in PBS and assayed for recognition by the different antibodies. All antibodies were expressed recombinantly as human IgG1. (**A**) Binding of PGT128/130 bnAb family members PGT125, PGT126, PGT128 and PGT130. (**B**) Binding of non-PGT128/130 family antibodies BF520.1, BG18, PCDN-33A, PGDM12, PGDM21, PCDN76-33A, VRC41.01. (**C**) Binding of inferred gl precursors BF520.1, BG18, PCDN-33A and the PGT128/130 family. Results are from single experiments performed with technical duplicates.

We first evaluated by ELISA the binding of oligomannose-specific bnAbs from the PGT128/130 family (PGT125, 126, 128, 130) to NIT211 (Ref.^6^), a CRM_197_-conjugated version of our lead oligomannose mimetic (Fig. 1). We had used these bnAbs previously for antigenic characterization of the BSA conjugate of the same glycoside^5^. All four antibodies bound NIT211 (4.1 glycans per CRM_197_) avidly (EC_50_ 0.1–7 nM; Fig. 2A). As reported recently^20^, these antibodies bind NIT211 at least as good as the BSA conjugate loaded at similar density.

**Figure 3:**
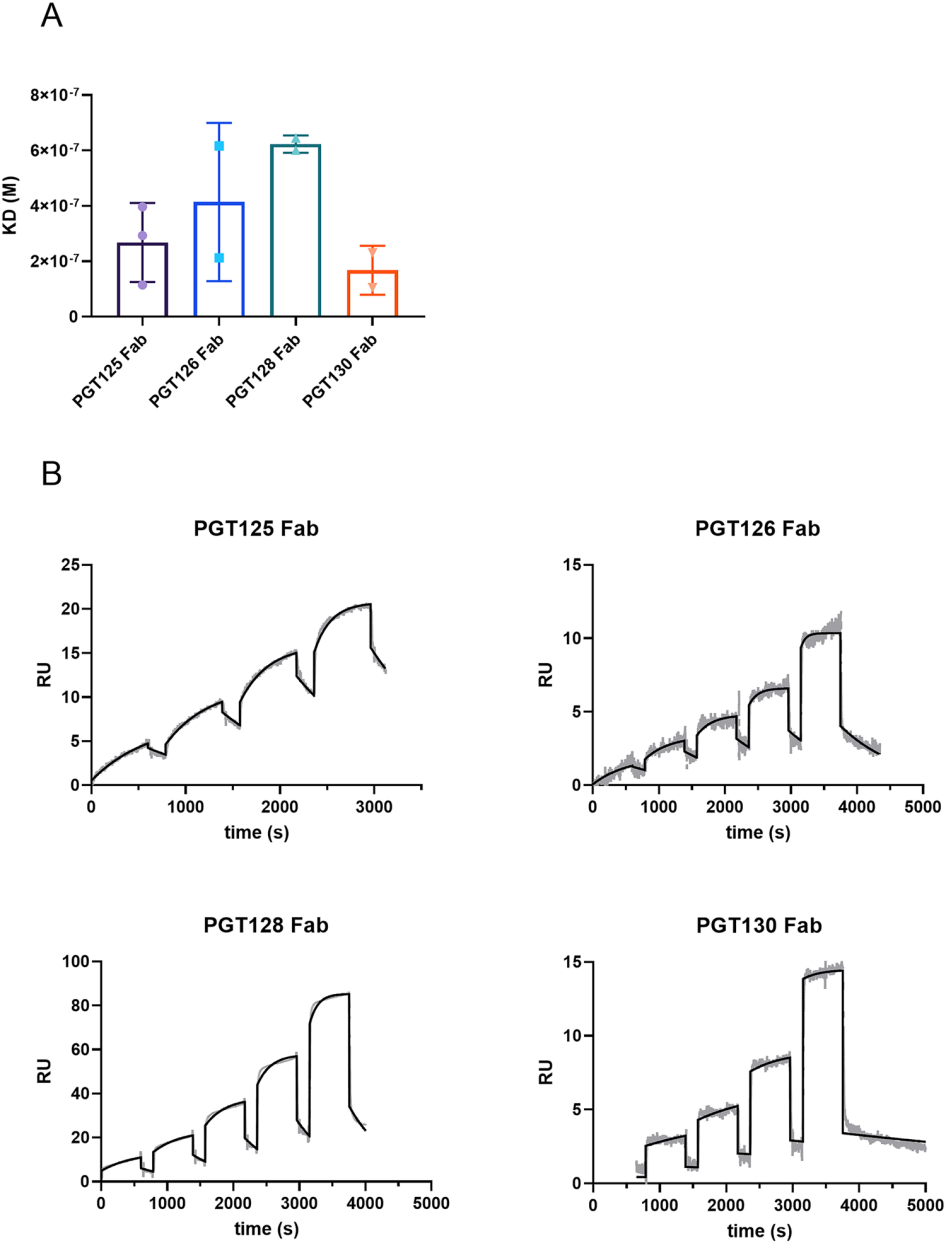
Binding affinities of PGT125, PGT126, PGT128 and PGT130 Fabs for NIT211 as measured by SPR. (**A**) Geometric mean of the association constant (ka), dissociation constant (kd) and equilibrium dissociation constant (K_D_) for the four PGT bnAbs. The results are from 2–3 independent experiments. (**B**) Representative sensorgrams of Fab binding to NIT211 using single-cycle kinetics. The calculcated association constant (ka), dissociation constant (kd) and K_D_ are indicated. The black lines show the data (grey) fitted to a 1:1 binding model.

We then used recombinantly expressed Fab fragments in conjunction with Biacore SPR to determine binding affinities of the four PGT antibodies for NIT211. A biotinylated version of NIT211 (4.1 glycans per CRM_197_), captured onto the sensor surface by immobilized streptavidin, served as the ligand. Results show that the Fabs bind NIT211 with average affinities ranging from 271 nM (PGT130) to 678 nM (PGT128) (Table 1, Fig. 3A). The K_D_ values determined here for PGT125 and PGT128 are similar to the reported monovalent K_D_ values of these antibodies for a Man_9_-V3 glycopeptide (PGT125: 706 nM; PGT128: 326 nM) and the estimated monovalent K_D_ of PGT128 for BG505 SOSIP (303 nM)^21^. Similar to others^22^, we observed biphasic sensorgrams for all the PGT antibodies despite the use of monovalent Fab molecules, suggestive of a bivalent binding interaction with NIT211 (Fig. 3B).

**Table 1:** Binding affinities of the Fab fragments of the four oligomannose-patch specific antibodies PGT125, PGT126, PGT128 and PGT130 for NIT211 as measured by Biacore SPR.

mAb (Fab fragment)	ka (M^-1^ s^-1^ × 10^3^) (sd)	kd (s^-1^ × 10^–3^) (sd)	K_D_ (M × 10^–7^) (sd)
PGT125	5.64 (2.44)	1.99 (2.05)	3.09 (2.03)
PGT126	4.9 (0.38)	1.97 (1.24)	4.14 (2.86)
PGT128	4.53 (2.71)	3.22 (2.33)	6.78 (1.09)
PGT130	1.28 (0.09)	0.37 (0.29)	2.71 (2.4)

mAb, monoclonal antibody; ka, association constant; kd, dissociation constant; K_D_, equilibrium constant; sd, standard deviation.

**Figure 4:**
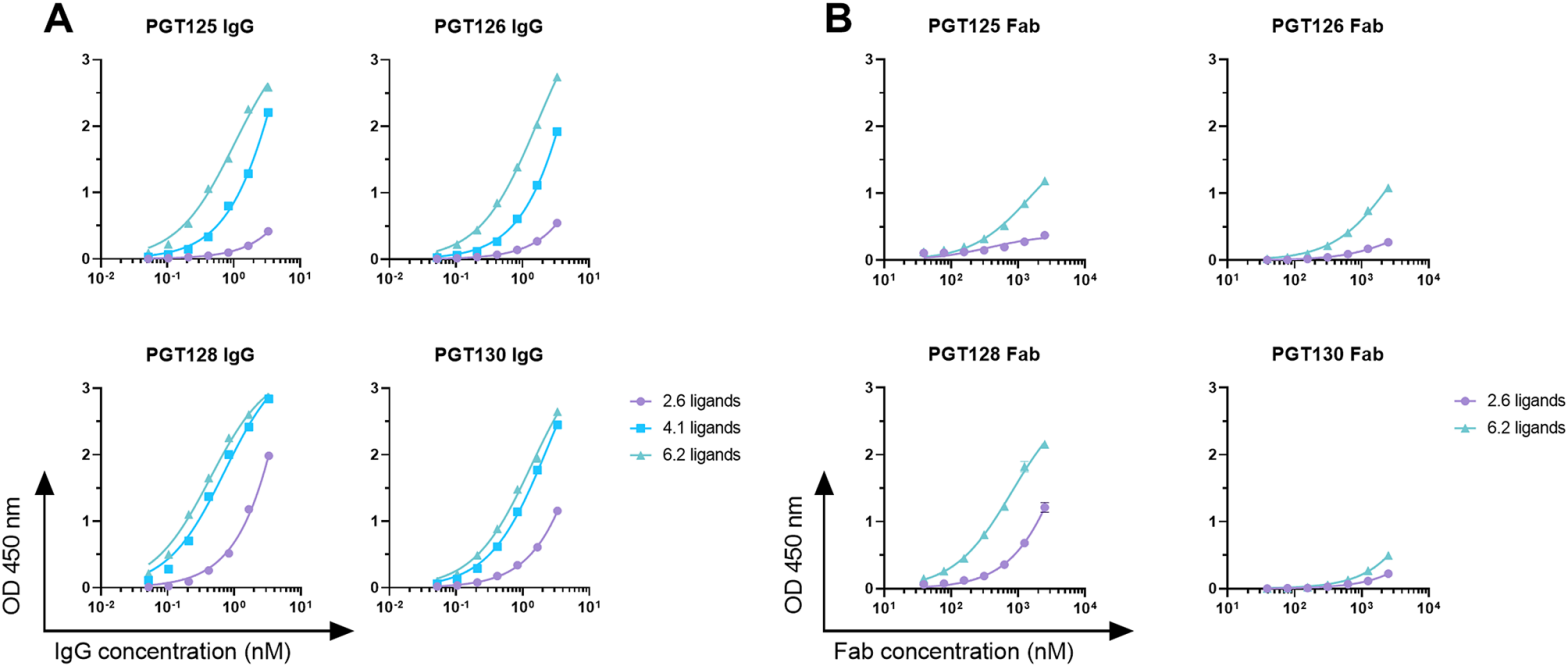
The relative binding affinities of PGT antibodies for NIT211 increase with increasing ligand density. Binding to NIT211 conjugates was assessed by ELISA. NIT211 (2.6 ligands, 4.1, and 6.2 ligands) was coated as solid-phase antigen onto ELISA plate wells at 5 µg/ml (82, 79, 76 nM respectively) and assayed for antibody binding. (**A**) Binding of PGT125, PGT126, PGT128 and PGT130 IgG to NIT211 at three different densities of glycoside per CRM_197_. (**B**) Binding of PGT125, PGT126, PGT128 and PGT130 Fabs to NIT211 at two different densities of glycoside per CRM_197_.

Oligomannose-specific bnAbs such as those from the PGT128/130 family interact with two or more glycans on HIV Env^23,24^. To evaluate the relationship between NIT211 glycan density and antibody binding avidity, and given the above-noted observations of biphasic antibody binding in SPR experiments, we assayed by ELISA binding of the PGT antibodies to NIT211 conjugated with the oligomannose mimetic at densities of 2.6, 4.1, and 6.2 glycans per CRM_197_. We first assessed binding of the antibodies formatted as IgGs. Consistent with our previous report^5^, antibodies of the PGT128/130 family bound increasingly avid to NIT211 with increasing glycan density (Fig. 4A), with apparent maxima at a density of ~ 6 glycosides. Strikingly, the relative binding affinity of the Fab fragments of antibodies PGT125, 126 and 128 also increased substantially upon glycan density (Fig. 4B) suggesting a bivalent or greater interaction of these Fab fragments to NIT211 ligands. Taken together with the SPR analyses, these results suggest that presentation of the oligomannose mimetic at a density of 4–6 molecules per CRM_197_ reasonably approximates oligomannose presentation on HIV gp120 conducive to the binding of at least the PGT128/130 family of bnAbs.

### Oligomannose patch-specific bnAbs outside of the PGT128/130 bnAb family bind less avidly to CRM_197_-glycoconjugate NIT211

Having established that our lead glycomimetic conjugated to CRM_197_ retains favorable antigenicity for members of the PGT128/130 bnAb family as compared to the BSA conjugate, we next evaluated binding of representatives from other bnAb families specific for the oligomannose patch (BF520.1, BG18, PCDN-33A, PGDM12, PGDM21, VRC41.01)^21,25–28^. We found that bnAbs BG18, PGDM21, and VRC41.01 bind NIT211 (4.1 glycans per CRM_197_) with reasonably high avidity (EC_50_ 14–70 nM) (Fig. 2B), albeit less than the PGT128/130 bnAbs. In contrast, bnAbs BF520.1, PCDN-33A and PGDM12 bound the CRM_197_ conjugate very poorly (EC_50_ 1–5 μM) (Fig. 2B). Binding of these antibodies as Fab fragments was not detectable by SPR. Overall, these results likely reflect the varied degrees of specificity of the bnAbs for oligomannose, their capacity to engage other glycans, and their relative dependence on interaction with the V3 protein backbone. Nevertheless, the results suggest that the oligomannose mimetic and its presentation on CRM_197_ sufficiently imitates the binding epitope of not just the PGT128/130 bnAb family but also a few other oligomannose-specific bnAbs.

### CRM_197_-conjugated glycoside mimic of oligomannose is bound also by inferred gl precursors of anti-HIV oligomannose-specific bnAb families

A prevailing thesis in the HIV vaccine field currently is that an immunogen that can be bound with sufficient affinity in vitro by gl precursors of existing bnAbs might be better able to activate the appropriate B cells in vivo to yield similar bnAbs (reviewed in ref.^1^). We therefore assessed the binding of published inferred gl precursors from four oligomannose-specific bnAb families (BF520.1, BG18, PCDN-33A and PGT128/130) for binding in ELISA to NIT211 (4.1 glycans per CRM_197_). As shown in Fig. 2C, all four gl antibodies bound NIT211, albeit with the expected lower avidity (EC_50_ 0.1–5 µM) compared to their mature cognates. Fab fragments of these gl antibodies did no bind detectably to NIT211 by SPR. Recognition of the conjugated oligomannose mimetic by various oligomannose bnAbs and, most importantly, some of their respective gl precursors provided encouragement in NIT211′s potential to trigger B cells with nominal specificity for oligomannose.

### CRM_197_-conjugated glycoside NIT211 elicits glycan-specific antibodies in human-antibody transgenic Trianni mice

Having determined that the NIT211 glycoconjugate is bound reasonably well by several oligomannose-specific bnAbs and a few gl versions thereof, we next sought to identify a possibly optimal adjuvant formulation for the elicitation of the desired glycan-specific antibody response. We chose to conduct our immunizations in the human-antibody transgenic Trianni mouse system to allow for an approximation of possible antibody responses in people. Before commencing, we confirmed that these mice do not develop an abnormal antibody response by subcutaneously administering a priming immunization with the model antigen KLH formulated in Alhydrogel plus CpG ODN1826. We found total IgG responses in Trianni mice to be as robust as similarly primed wild‐type C57BL/6 mice at one month post-immunization (Supplementary Fig. 1A), albeit that the IgG1 and IgG2c responses in Trianni were notably stronger (Supplementary Fig. 1B). Total T‐follicular helper (Tfh) cell frequencies (B220^-^CD4^+^CD44^hi^PD1^+^CXCR5^+^) in draining inguinal lymph nodes at day 8 post prime (4.9–13.1%) were also similar to the average frequencies reported in the draining popliteal lymph nodes of non-transgenic mice^29^ after a single footpad immunization with KLH formulated with alum (Supplementary Fig. 2).

We then assessed and compared the immunogenicity of NIT211 formulated in three different types of adjuvants: Alhydrogel, AddaVax, and GLA-SE. The NIT211 conjugate used for immunization carried an average of 6.2 glycosides per CRM_197_ molecule, which, as shown in Fig. 4A, was bound strongly by the PGT antibodies. Mice (n = 5 per group) were primed at day 0 and boosted at days 21, 42 and 105, a schedule modeled on glycoconjugate vaccine schedules in humans^30,31^, and sera collected on days 0, 10, 28, 49, and 119.

All mice immunized with the GLA-SE formulation mounted a rapid IgG response to the CRM_197_ carrier protein by day 10 after priming (Supplementary Fig. 3), consistent with previous reports^20^ and the expected high immunogenicity of the carrier. The magnitude of the antibody response was somewhat slower in mice immunized with the Alhydrogel and AddaVax formulations. Even so, IgG levels in all three animal groups plateaued following the second boost (day 49; Supplementary Fig. 3). Overall, mice immunized with the GLA-SE formulation produced the highest IgG response to the CRM_197_ carrier protein, followed by the AddaVax and Alhydrogel groups, respectively.

**Figure 5:**
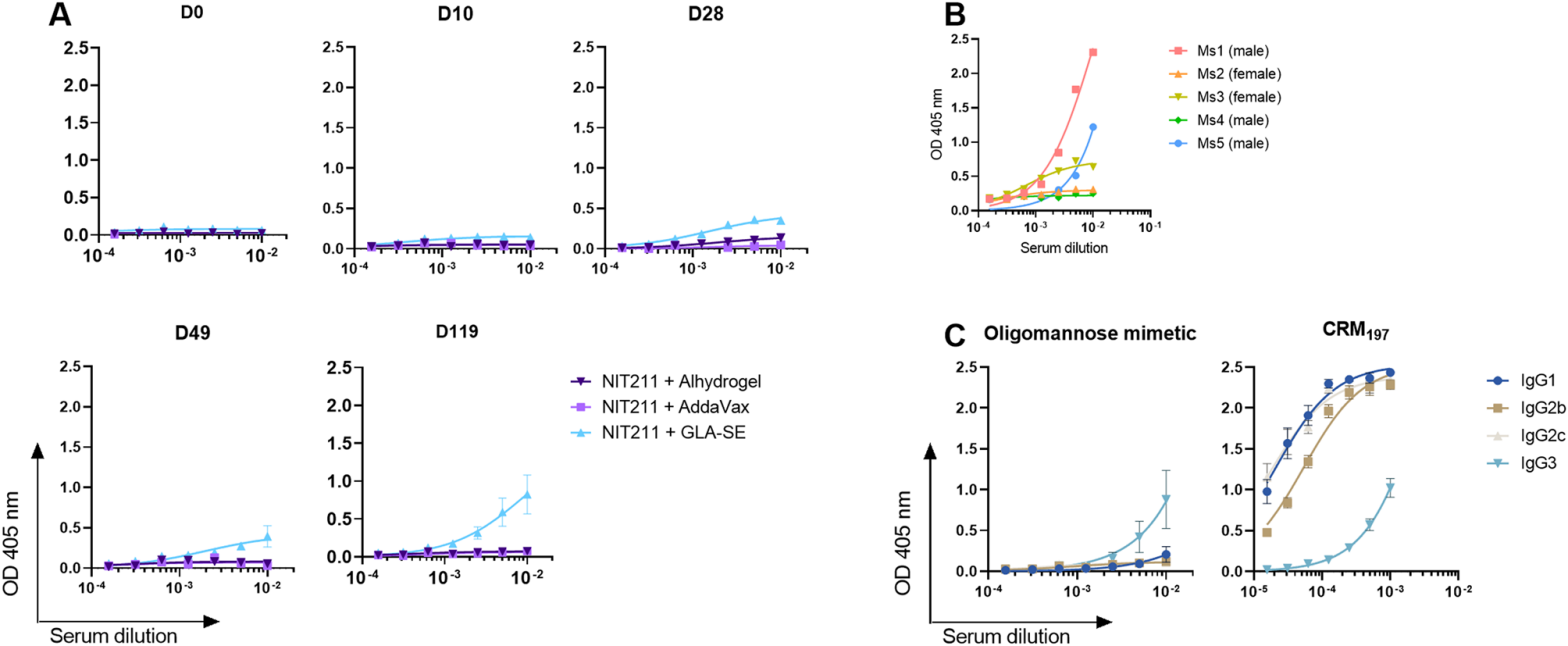
Only animals administered GLA-SE-adjuvanted NIT211 mount an IgG response to the oligomannose mimetic that is of the IgG3 subclass. Trianni mice (n = 5 per group) were immunized subcutaneously (days 0, 21, 42 and 105) and sera collected on day 0 prior to immunization and on days 10, 28, 49, and 119 post-immunization. (**A**) Binding of total IgG antibodies in pre-immune and post-immune sera to BSA-conjugated oligomannose mimetic. Binding curves represent mean values for the five animals in each immunization group, each tested in duplicate, with error bars denoting the standard deviation from the mean. (**B**) Individual IgG binding curves for NIT211 + GLA-SE immunized mice (Ms1 to 5). The sex of each mouse is also noted. (**C**) IgG1, IgG2b, IgG2c and IgG3 antibody subclass responses in day 119 post-immune sera of NIT211 + GLA-SE immunized animals for the BSA-conjugated oligomannose mimetic in comparison to the CRM_197_ protein carrier.

To measure antibody responses to the oligomannose mimetic without interference from antibodies specific for the CRM_197_ carrier, we used the BSA-conjugated version of the glycoside (dubbed NIT82b^5^), which was conjugated at 4 glycosides per BSA molecule. We confirmed that none of the sera from the final bleed (day 119) bound unconjugated BSA (Supplementary Fig. 4), meaning that any measured binding to NIT82b should be due to the conjugated glycoside. Unexpectedly, we found that only mice immunized with the GLA-SE adjuvanted conjugate produced IgG that bound the oligomannose mimetic as presented on BSA (Fig. 5A). We found no evidence of glycoside-specific IgM antibodies in sera from any of the three groups of immunized animals (Supplementary Fig. 5). The level of IgG binding to the BSA conjugate varied among the GLA-SE-immunized mice; serum antibodies from two of the animals bound reasonably strongly, serum from a third bound at modest levels and serum from the two remaining animals bound poorly (Fig. 5B). The reason for this heterogeneity is not immediately obvious. One possibility is that antibodies elicited in some animals may not be able to bind sufficiently avid to particular configurations of glycoside molecules as presented on the BSA-conjugated version of our mimetic.

Strikingly, IgG3 was the most prevalent IgG subclass in the response of GLA-SE immunized animals to the oligomannose mimetic, whereas IgG1 and IgG2 (IgG2b and IgG2c) were the most prevalent against the CRM_197_ carrier (Fig. 5C).

In sum, the results above show that NIT211 formulated in GLA-SE induced the greatest antibody response to the glycomimetic compared to formulations with Alhydrogel or Addavax.

### Anti-glycan serum antibodies elicited by CRM_197_-glycoconjugate NIT211 in GLA-SE bind recombinant HIV gp140 trimers but are unable to exert neutralizing activity

**Figure 6:**
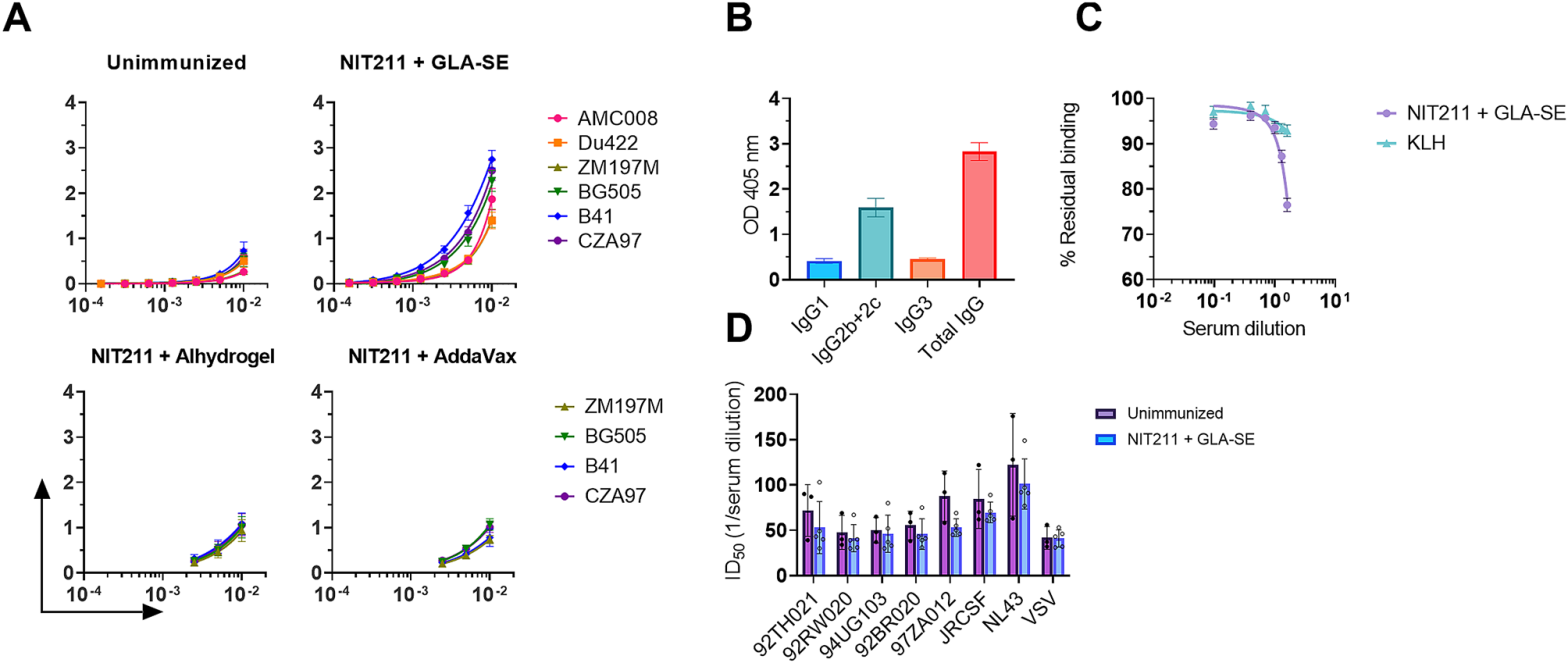
NIT211 formulated in GLA-SE elicits antibodies with capacity to bind recombinant HIV Env trimers. (**A**) Binding of total IgG from day 119 sera from unimmunized, and NIT211-immunized animal groups to SOSIP trimers AMC008 (subtype B), Du422 (subtype C), ZM197M (subtype C), BG505 (subtype A), B41 (subtype B), and CZA97 (subtype C). The HIS-tagged trimers were captured on nickel-coated ELISA plates at 5 µg/ml in PBS. (**B**) Level of IgG1, IgG2, IgG3 antibody subclass binding (1:100 dilution) to B41 SOSIP trimer in day 119 sera of NIT211 + GLA-SE immunized animals. (**C**) Residual binding of bnAb PGT128 to B41 SOSIP trimer following incubation with sera from NIT211 + GLA-SE immunized animals (day 119) vs sera from KLH + Alum/CpG ODN1826 immunized animals (day 34). (**D**) Day 119 sera from NIT211 + GLA-SE immunized animals and sera from a group of unimmunized animals were assessed for pseudovirus neutralization using a panel of seven diverse HIV-1 strains (92TH021, 92RW020, 94UG103, 92BR020, 97ZA012, JRCSF and NL4-3). Pseudotyped vesicular stomatitis virus (VSV) was used as a negative control. All graphs depict mean values for the serum samples from all animals (n = 5) in each group. Error bars represent standard error from the mean.

We next assessed whether the elicited anti-glycan antibody response could recognize glycans on HIV. First, we measured serum IgG binding to a panel of SOSIP-based HIV gp140 trimers by ELISA. Notable IgG binding was observed with the sera of all five animals in the GLA-SE group (Fig. 6A), including sera from the two animals that had bound only marginally to the BSA conjugate. This unexpected result prompted us to also assay sera from the animals immunized with the Alhydrogel and Addavax formulations. Sera from these animals bound meagerly to a selection of SOSIP trimers and this binding was similar to the binding of sera from unimmunized mice (Fig. 6A), which we assume is caused by low levels of naturally occurring anti-glycan antibodies in serum. Using day 0 pre-immunization sera from the GLA-SE group, we confirmed that antibody binding to SOSIP trimers was not due to pre-existing anti-glycan IgG antibodies (Supplementary Fig. 6).

IgG subclass analyses revealed that whereas IgG3 was the predominant IgG subclass responsible for binding to the oligomannose mimetic in sera from animals immunized with the GLA-SE formulation (cf. Figure 5C), serum antibodies binding to the SOSIP trimers were predominantly IgG2 (Fig. 5B). These results suggest that the immunization with NIT211 yielded two different anti-glycan responses; one, mainly of the IgG3 subclass, specific only for the synthetic glycoside, and the other, mainly of the IgG2 subclass, capable of binding glycans on recombinant HIV Env trimers.

To determine whether the NIT211 + GLA-SE formulation had truly stimulated antibodies with capacity to bind glycans within or at least near the high-mannose patch on HIV gp120, we performed an inhibition ELISA assay in which we measured the binding of bnAb PGT128 to B41 SOSIP trimer after incubation with GLA-SE sera vs immune sera from animals immunized with KLH, which itself carries a large number of high mannose-type glycans^32^. We observed a decrease in PGT128 residual binding following incubation with the NIT211 + GLA-SE sera compared to KLH immune sera (Fig. 6C). These results suggest that NIT211 formulated with GLA-SE had indeed stimulated the production antibodies, at least in some animals, capable of binding glycans within or neighboring the high-mannose patch. More detailed mapping, planned for future studies, will be needed to confirm this notion.

Given the observed binding of the NIT211 + GLA-SE sera to SOSIP trimers, we assessed the sera for neutralizing activity against a small panel of seven HIV-1 strains from different subtypes in the Monogram PhenoSense neutralization assay. We confirmed (Supplementary Table S1) that, with the exception of HIV NL4-3, all chosen viruses are highly sensitive to neutralization of affinity matured PGT128 and PGT130 (ref.^33^). We determined also that none of the viruses are neutralized by the inferred gl precursor of PGT128/130 but that one is neutralized by an early precursor (dubbed 3L74H) of the PGT128/130 family (Supplementary Table S1). Disappointingly, we observed no meaningful neutralizing activity relative to unimmunized animals even at the lowest serum dilution tested (1:30) (Fig. 6D), suggesting that any activity measured was likely non-specific and not related to the immunization (i.e., anti-glycan antibodies elicited). Moreover, we observed no statistical difference in average ID_50_ between the negative control virus (VSV), which lacks oligomannose, and most of the HIV strains assayed, further supporting the notion that for these HIV strains any activity was likely due to non-specific serum factors. The only exception was virus NL4-3, derived from a lab-adapted HIV strain. NL4-3 was however neutralized equally by sera from unimmunized and immunized animals, suggesting that the observed neutralizing activity was not due to antibodies elicited from immunization. We concluded from these results that the elicited antibodies are of insufficient affinity (or avidity) to bind glycans on the virus surface or at least not enough to exert neutralizing activity.

## Discussion

In this study we have identified the TLR4 agonist adjuvant GLA-SE as conducive to the elicitation of glycan-specific HIV-binding antibodies by NIT211, a CRM_197_ conjugated oligomannose mimetic. Of note is that we were able to elicit the aforementioned antibodies in animals expressing a full human antibody repertoire, demonstrating the ability of the presented mimetic to be sensed by naïve B cells of the desired specificity in a milieu that is not necessarily favorable. Although the level of the elicited anti-glycan antibody response was insufficient to exert meaningful neutralizing activity, we consider the ability of the elicited antibodies to bind native-like HIV trimers promising. Whether the ability of serum antibodies to bind SOSIP trimers but not exert neutralizing activity relates to the somewhat higher proportion of oligomannose on recombinant trimers compared to native functional Env trimers on the virus^34^ will require further investigation.

Our analyses of the antigenic characteristics of NIT211 led to several notable observations. First, the apparent binding affinity of PGT128 and several related antibodies for our neoglycoconjugate increased with increasing glycan density (Fig. 4), which suggests that increased glycan density results in the presentation of oligomannosides in proximity to a peptide backbone that may resemble the cognate epitope of the PGT antibodies better and therefore enhance antibody binding. Analogous outcomes have been observed using tetramannosides conjugated to BSA and anti-HIV antibody 2G12, with increased antibody binding correlating with increased glycan density^35^. The close binding affinity of Fab PGT128 for NIT211 (0.6 μM; Fig. 3) and its estimated affinity for SOSIP gp140 (0.3 μM^21^) suggests that presentation of the mimetic on CRM_197_ at least somewhat imitates the organization of oligomannose residues on Env to which PGT128 and related antibodies bind.

In addition to strong binding by members of the PGT128/130 family, we also observed reasonably avid binding by the oligomannose-specific bnAbs VRC41.01, PGDM21 and BG18 (Fig. 2B), suggesting that presentation of the mimetic on CRM_197_ also somewhat resembles their respective cognate epitopes on HIV Env. However, other antibodies (BF520.01, PGDM12 and PCDN-33A) bound poorly. Different factors may be limiting the ability of these antibodies to bind more avidly. For example, the glycan binding profile of VRC41.01^21^ suggests that this antibody binds an epitope involving the reducing end of *N*-linked oligomannose. The lack of a chitobiose core at the reducing end of our mimetic may be one reason for VRC41.01′s inability to bind the mimetic more avidly. Another example is BG18, which binds a complex set of 3–4 oligomannose-type glycans on Env^36^ that is unlikely to be fully replicated on NIT211, thus perhaps explaining that antibody’s inability to bind more avidly to the glycoconjugate. In contrast, PCDN antibodies such as PCDN-33A have been shown to bind only to Man_9_GlcNAc_2_^27^, indicating strong dependence on the D2 arm of oligomannose—which is not replicated in the mimetic and therefore likely explains the poor binding of that antibody.

NIT211′s potential for triggering the elicitation of oligomannose-specific antibodies is evident also from the binding of inferred gl precursors from several of the oligomannose-specific bnAbs that were assayed (BF520.1, BG18, PCDN-33A, PGT128/130) (Fig. 2C). We did note that binding of the PGT128/130 gl antibody was the least of all the antibodies assayed (EC_50_ 1.3 µM). Given the strong binding of affinity matured descendants such as PGT128, these results underscore the extent to which this family of antibodies has evolved to gain affinity for oligomannose while maintaining promiscuity for different oligomannose structures^33^. In notable contrast, binding of the PCDN-33A gl antibody was the strongest of the four gl antibodies assayed (EC_50_ 0.2 µM), yet binding of the affinity matured descendant was poor (Fig. 2B). PCDN-33A’s binding specificity for full-sized oligomannose (Man_9_)^27^ suggests that as the gl antibody matured, it lost its relative promiscuity by mutating away from being able to recognize lower-order oligomannose structures.

Analyses of NIT211′s immunogenicity also led to in a few notable and unexpected observations. First, although we found that the GLA-SE formulation yielded a balanced IgG1/IgG2 antibody response to the CRM_197_ carrier protein as expected^17^, we were struck by the IgG3 subclass response to the glycoside component (Fig. 5). This distinctive response is noteworthy given that IgG3 is a common subclass among bacterial oligosaccharide-specific antibody responses in mice^37–39^ and given that we designed our mimetic to appear foreign to the immune system^5^. We therefore interpreted these results as suggesting that the Trianni mouse immune system sensed our glycoconjugate as a bacterial polysaccharide, possibly aided by its formulation in a TLR4 agonist adjuvant. Unexpectedly however, these IgG3 antibodies appear not responsible for binding to HIV-1 SOSIP trimers. Instead, our analyses revealed that a second glycan-specific antibody response, apparently of the IgG2 subclass, was induced that is able to bind SOSIP trimers (Fig. 6). Whether these subclass-distinctive responses developed independently parallel to each other or whether one developed after the other requires further investigation, as this may reveal an immunization time point at which to possibly boost the HIV-cross reactive antibody response. Nevertheless, as discussed further below, possible trimming of our glycoside mimetic by a soluble mannosidase during the first immunizations gives reason to believe that HIV-cross reactive antibodies likely developed later in the immunization process.

We interpret the difference between the SOSIP-binding IgG2 antibodies and the oligomimetic-binding IgG3 antibodies as being caused by the so-called D2 arm in oligomannose. Presumably, the elicited IgG3 antibodies are unable to accommodate the D2 arm, which is absent in the mimetic, and therefore do not bind the SOSIP trimers. Conversely, the IgG2 antibodies are presumed as able to accommodate the D2 arm. Given that the mimetic lacks the D2 arm, the IgG2 antibodies likely do not bind the D2 arm; rather, they presumably bind in a manner that is not impeded by the D2 arm on natural oligomannose.

How then to explain our inability to measure IgG2 antibodies to the mimetic? One possibility is that the glycoside configuration on the BSA conjugate, used to assess antibody responses to the oligomannose mimetic without interference from carrier-specific antibodies, allows for more avid IgG3 binding compared to IgG2. As reported elsewhere^20^, several of the oligomannose-specific PGT antibodies (PGT125, 126 and 130) bind the CRM_197_ conjugate better than the BSA conjugate, suggesting differences in glycoside configuration. The BSA conjugate may thus allow for mouse IgG3 binding but not IgG2. We recognize however that further investigation, for example using antibodies made with the backbone of different IgG subclasses, will be needed to satisfy this seeming conundrum.

Some HIV bnAbs may exhibit auto- and/or polyreactivity^40^. However, given the modest levels of anti-glycan antibody responses elicited here, we have not explored whether sera from animals immunized with the NIT211 + GLA-SE formulation exhibit auto- or polyreactivity. Nevertheless, we received no report of any of the immunized animals suffering from health issues, suggesting that self-reactive antibodies in these animals, if any, do not have any meaningful or otherwise apparent physiological impact.

There is general belief that at least one major difficulty in eliciting oligomannose-specific bnAbs stems from B cell tolerance (reviewed in^41^). Synthetic oligomannose-like glycosides, such as the ones that we are pursuing^5,6,42^, are one potential avenue to overcome tolerance, based on the notion that anergic B cells can be ‘roused’ by exposing them to seemingly cross-reactive antigens along with the proper immunostimulatory signals^43^. However, a thesis put forth recently by Nguyen et al.^44^ suggests that trimming of oligomannose-based conjugates upon immunization, perhaps rather than immune tolerance, may be impeding the elicitation of oligomannose-specific bnAbs. As published recently, our own investigations revealed that after prime and two boosters, sera of mice immunized with NIT211 + GLA-SE produced an IgG response that bound better to serum mannosidase-treated (i.e., trimmed) conjugate compared to the buffer-treated conjugate^20^. This might explain our inability to measure much of a response to the target glycoside until after the second booster (day 49; Fig. 5). We found however that sera from NIT211 + GLA-SE immunized mice generally exhibited less preference for mannosidase-trimmed glycoside by the end of the immunization series (Day 119; Supplementary Fig. 7A). These observations suggest that the immunization scheme used here allowed trimming of the administered glycoside by soluble mannosidase in vivo to be mitigated. Nevertheless, it will be of interest to determine whether a mimetic protected from mannosidase trimming yields greater HIV-reactive antibodies.

Another group^45^ has posited that nAb induction might be impaired due to the action of mannose-binding lectin (MBL). We did not determine MBL levels in the Trianni mice used here and therefore cannot fully exclude this possibility for our glycoconjugate. However, our ability to evoke glycan-specific antibodies, including some with the capacity to bind glycans on recombinant HIV trimers, suggests that hindrance by MBL, if any, is not absolute. One possible explanation is that the MBL acute phase response in laboratory mice appears to peak already at ~ 30 h^46^, which may allow enough immunogen to escape elimination by MBL.

In conclusion, we show here that our CRM_197_-conjugated oligomannose mimetic exhibits promising immunogenicity, as shown by the elicitation of antibodies that can bind glycans on soluble recombinant trimers. Although these antibodies did not exert neutralization activity, our analyses help to inform on the kinetics of the desired response. An obvious next step is, for example, to evaluate whether boosting with a SOSIP trimer can generate higher-affinity antibodies.

## Experimental methods

### Glycoside synthesis and neoglycoconjugate preparation

The oligomannose mimetic was synthetized as previously described^5^. The BSA conjugate of the ligand, termed NIT82b, with an average loading density of 4 glycosides per BSA molecule, was also prepared as previously reported^5^. The CRM_197_ conjugates, collectively dubbed NIT211, at densities ranging from 2–6 glycosides per CRM_197_ molecule, were synthesized as described elsewhere recently^20^. Biotinylated NIT211 was generated with a sulfo-NHS biotinylation kit (ThermoFisher) in the same manner as reported previously for the BSA conjugate^5^.

### Recombinant antibody expression and purification

PGT125, 126 128 and 130 Fabs were expressed in FreeStyle 293S cells (Life Technologies) grown in suspension by transfection at a 1:1 ratio of plasmids encoding light and heavy chain (truncated at Asp^H234^). Supernatants harvested 6 days after transfection were passed over an anti-human lambda affinity matrix (CaptureSelect LC-lambda (hu); Thermo Scientific) equilibrated in PBS. Fab fragments were eluted with 0.1 M glycine, pH 3.0 and neutralized with 10 × concentrated PBS. Recombinant IgGs of all antibodies used in this study were expressed in FreeStyle 293 F cells and purified on a protein A resin (Thermo Scientific) using the aforementioned glycine solution for elution, as recently described^20^.

### Animal immunizations

Trianni mice (6–8 weeks of age at the start of immunization) were immunized under contract at Antibody Solutions (Santa Clara, CA). The animals were obtained from Charles River Labs (Wilmington, MA) and randomly divided into groups of 5 animals, with a roughly equal mix of male and female animals in each group to minimize effects due to biological sex. Animals (n = 5 per group) were immunized subcutaneously (base of tail) with NIT211 conjugate formulated in 2% Alhydrogel (Invivogen), AddaVax (Invivogen) or GLA-SE (IDRI). All immunizations were performed in an unblinded manner. We utilized the Resource equation method^47^ (E = N-T, with 10 < E < 20 and N = n-1) to determine that 5 animals are adequate for comparing three or more groups. Anesthesia (isoflurane) was used to sedate animals during injections. Mice were primed at day 0, and boosted at day 21, 42 and 105. Each mouse received 30 µg of glycoconjugate, corresponding to ~ 3 µg of carbohydrate. Alhydrogel was mixed with antigen at a ratio of 2.5:1 (v/w), AddaVax with an equal volume of antigen, as per manufacturer’s instructions, and GLA-SE was prepared by mixing each dose of antigen with 20 μl each of an oil emulsion and a GLA emulsion, resulting in a dose of 5 µg of GLA per animal per dose. All formulations were diluted in endotoxin-free TBS. The immunizations were approved by Simon Fraser University’s Animal Care Committee (protocol. no. 1242HS-17) and all animal experiments were performed in accordance with institutional guidelines and regulations. No notable adverse reactions were observed. A small bleed (< 10% maximum blood volume) was collected via the saphenous vein from all animals just prior to immunization on day 0, at day 10 post-prime, and then on days 28 and 49 after the first and second booster injections, respectively. The animals were exsanguinated following euthanasia on day 119, two weeks after the third booster injections, and splenectomized. Euthanasia was done by CO_2_ inhalation followed by cervical dislocation. All collected blood was left to clot and serum recovered, which was then frozen until required for analyses. Splenocytes were recovered from the collected spleens using Histopaque-1077. An additional group of 5 mice served as unimmunized controls and sampled in analogous manner as described above for immunized animals. No animals or animal groups were excluded from any of our analyses, which were conducted in an unblinded manner. The study was carried out in compliance with the ARRIVE guidelines (https://arriveguidelines.org/).

## ELISA

To assess binding of oligomannose-specific bnAbs and serum antibodies to the NIT211 glycoconjugate, the antigen was absorbed onto 96-well polystyrene ELISA plates (Corning) at 5 µg/ml in PBS overnight at 4 °C. After washing, the plates were blocked with PBS supplemented with 3% (w/v) casein (PBS-C). BnAbs and immune sera were serially diluted in PBS-C plus 0.02% (v/v) Tween-20 (PBS-C-T). After washing, whole IgG bnAbs were detected with horseradish peroxidase (HRP)-conjugated anti-human Fc IgG (Jackson ImmunoResearch) and 3,3′,5,5′-tetramethylbenzidine (TMB) (Ultra TMB, Thermo Scientific). Reactions were stopped after 5 min with 2 M sulfuric acid and absorbances measured immediately at 450 nm. Fabs were detected with CaptureSelect™ biotinylated anti-human CH1 antibody (Thermo Scientific; 0.2 µg/ml in PBS-C-T) in conjunction with alkaline phosphatase conjugated streptavidin (Jackson ImmunoResearch; 0.6 µg/ml in PBS-C-T) and nitrophenylphosphate substrate (Sigma). Reaction were developed for 30 min and absorbances measured immediately thereafter at 405 nm. Total serum IgG was detected with a mixture of equal amounts of alkaline phosphatase conjugated anti-mouse IgG1, IgG2b, IgG2c and IgG3 secondary antibodies (Jackson ImmunoResearch) and nitrophenylphosphate substrate (Sigma). For detecting individual subclasses, the aforementioned secondary antibodies were used alone. Reactions were allowed to develop for 30 min, after which absorbances were measured at 405 nm. Serum mannosidase trimming experiments were performed as described elsewhere^20^.

To assess serum binding to SOSIP trimers, purified HIS-tagged trimers (kindly provided by John Moore (Weill Cornell Medical College) and Rogier Sanders (Amsterdam UMC)) were absorbed onto nickel-coated 96-well ELISA plates (Thermo Scientific) at 5 µg/ml in PBS overnight at 4ºC and then blocked with PBS-C. The remaining steps for detection of serum antibody binding were performed as described above.

For inhibition ELISAs, B41 SOSIP.v4.1 HIS-tagged trimer was absorbed onto nickel-coated 96-well ELISA plates as described above. After blocking with PBS-C, sera were serially titrated in PBS-C-T onto the bound antigen. After washing, PGT128 was added at fixed concentration (0.5 µg/ml in PBS-C-T). After incubation (1 h) and washing, PGT128 residual binding was detected with HRP-conjugated secondary antibody and TMB substrate as described above.

### Antibody affinity measurements

Antibody binding affinities were determined by surface plasmon resonance (Biacore X100) using Fab fragments and single cycle kinetics analysis. Biotinylated NIT211 (4.1 glycosides) was captured onto a sensor chip pre-immobilized with streptavidin using the biotin CAPture kit (400 RU). Fab fragment binding was determined usually serially titrated antibody, starting from 2.5 µM. All resonance signals were corrected by double referencing; signals monitored on the binding active channel were subtracted from the reference channel (sensor surface not modified with any reference protein) and by buffer signals. Data were globally fit to a 1:1 binding model within the BIA software.

### Pseudovirus-based neutralization assays

Sera were tested for virus neutralizing activity under contract at Monogram Biosciences (South San Francisco, CA) their pseudovirus-based neutralization assay with U87 target cells expressing CD4, CCR5, and CXCR4 as previously described^48,49^. A small panel of six HIV-1 strains (92TH021, 92RW020, 94UG103, 92BR020, 97ZA012, JRCSF) were assayed, chosen for their varied sensitivity to oligomannose-specific bnAbs^50^. The ultra-sensitive HIV NL4-3 strain, which is derived from a lab-adapted virus isolate, was included also. Vesicular stomatitis virus G pseudotyped virus was used as a negative control because the VSV G glycoprotein does not express oligomannose on its surface^51^. The sera were assayed at a starting dilution of 1/30. Serum neutralization titers were considered significant only if ≥ 3 times greater than the values obtained for the negative control virus.

### Statistics

Data were analysed using GraphPad Prism 9.0 software (San Diego, CA) and, unless otherwise stated, are presented as the mean with error bars denoting the standard error from the mean. Data for antibody binding experiments are from two or three experimental replicates. ELISA EC_50_ values were determined with GraphPad Prism 9.0 software using the one site specific binding equation model after subtraction of values for blank readings. Serum antibody analyses represent variance among animals (5 per group) in a single experiment.

## Supplementary Information

Supplementary Information
