# Supplementary material for: A glycoside analog of mammalian oligomannose formulated with a TLR4-stimulating adjuvant elicits HIV-1 cross-reactive antibodies
Source: Sci Rep. 2021 Feb 25;11:4637. doi: 10.1038/s41598-021-84116-w (PMC7907241; doi:10.1038/s41598-021-84116-w)
Supplement: Supplementary file 1 — Supplementary Information [file 41598_2021_84116_MOESM1_ESM.docx]

**SUPPLEMENTAL INFORMATION**

**A glycoside analog of mammalian oligomannose formulated with a TLR4-stimulating adjuvant elicits HIV-1 cross-reactive antibodies**

Jean-François Bruxelle, Tess Kirilenko, Nino Trattnig, Yiqiu Yang, Matteo Cattin, Paul Kosma, Ralph Pantophlet*

*Correspondence and requests for materials should be addressed to:

R.P. ([rpantophlet@sfu.ca](file:///\\fhs-file.fhs.sfu.ca\Shared\Research%20Staff\RP%20Pantophlet%20Lab\Current%20Lab%20Members\Jean-François%20Bruxelle%20(JFB)\Manuscripts\Nature%20communications\rpantophlet@sfu.ca))

**SUPPLEMENTARY FIGURES**

**
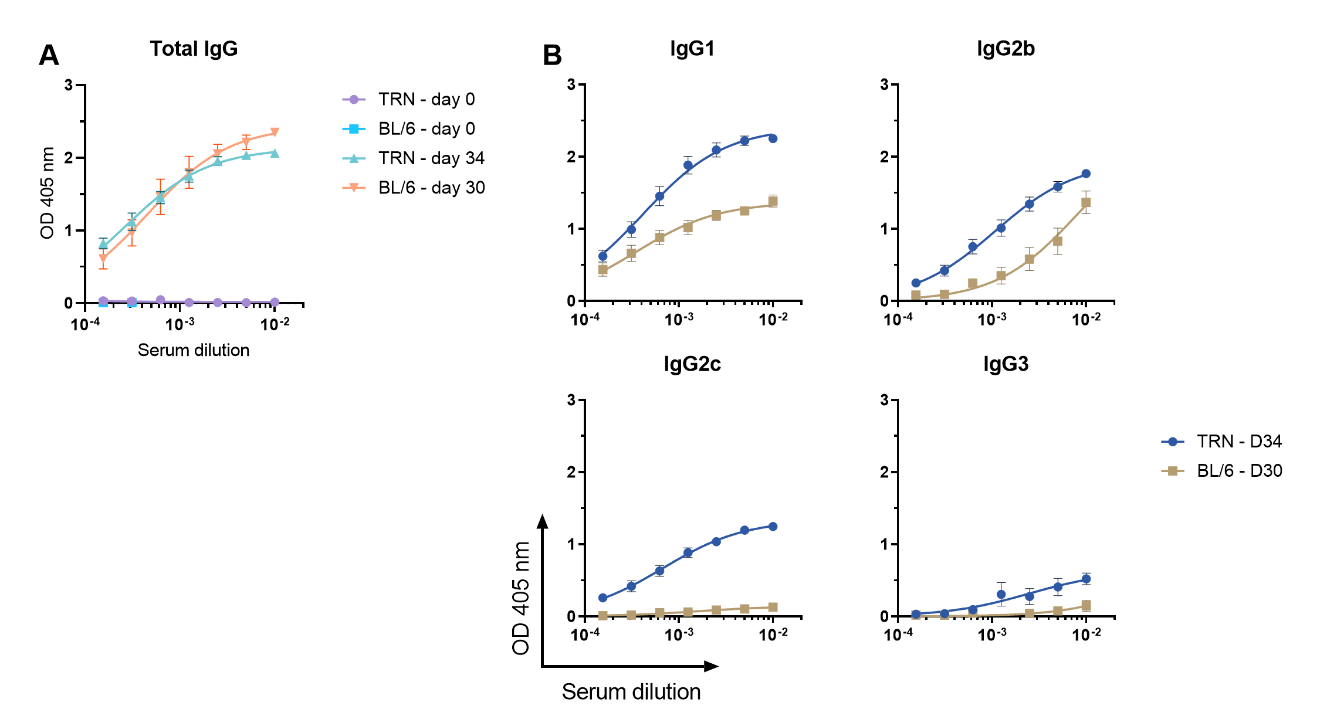
**

**Supplementary Figure 1. Prime immunization of Trianni mice, induces a robust antigen-specific IgG response.** (A) Binding of total IgG from pre-immune sera (n=3 per animal group) collected on day 0 and post-immune sera collected at day 30 (BL/6 mice; n=3) or day 34 (Trianni mice; n=7) to KLH. (B) Binding of IgG1, IgG2b, IgG2c, IgG3 antibody in post-immune sera to KLH. Graphs depict the mean values for the assayed serum samples, each tested in duplicate, with error bars denoting the standard deviation from the mean.


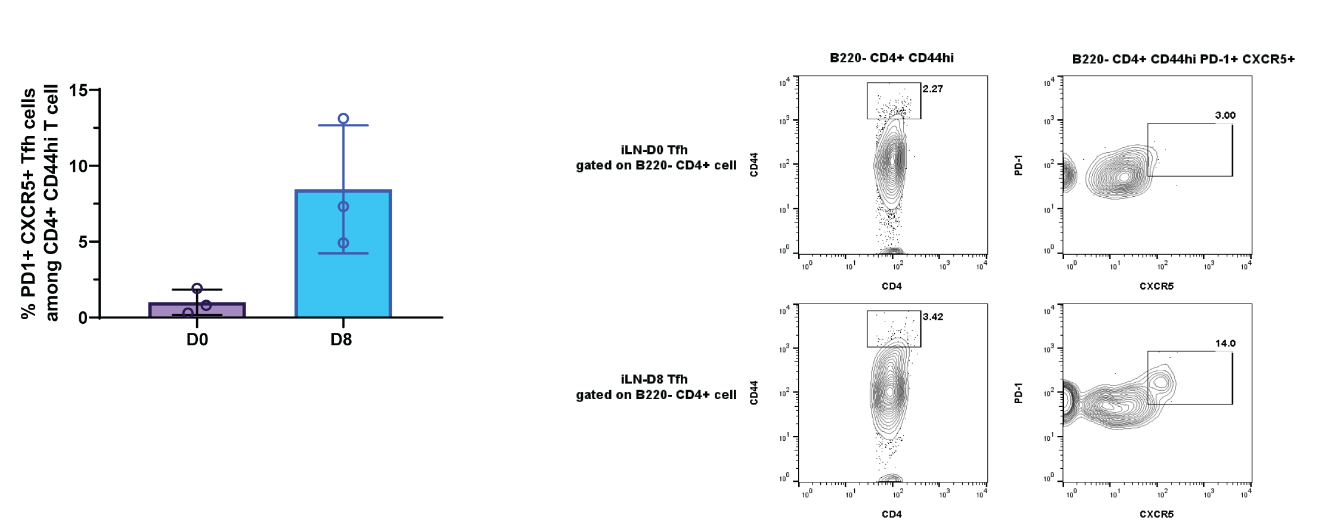


**Supplementary Figure 2. Trianni mice develop adequate germinal center Tfh responses in draining lymph nodes after a priming immunization with a model antigen.** Inguinal lymph nodes on day 8 post-prime of Trianni mice (n=3) injected with KLH formulated in Alhydrogel plus CpG ODN1826 were collected and analyzed by flow cytometry for Tfh cell frequencies (B220^-^CD4^+^CD44^hi^PD-1^+^ CXCR5^+^). Bar graphs depict the average of 3 mice. On the right, flow cytometry plots representative for each group is shown.


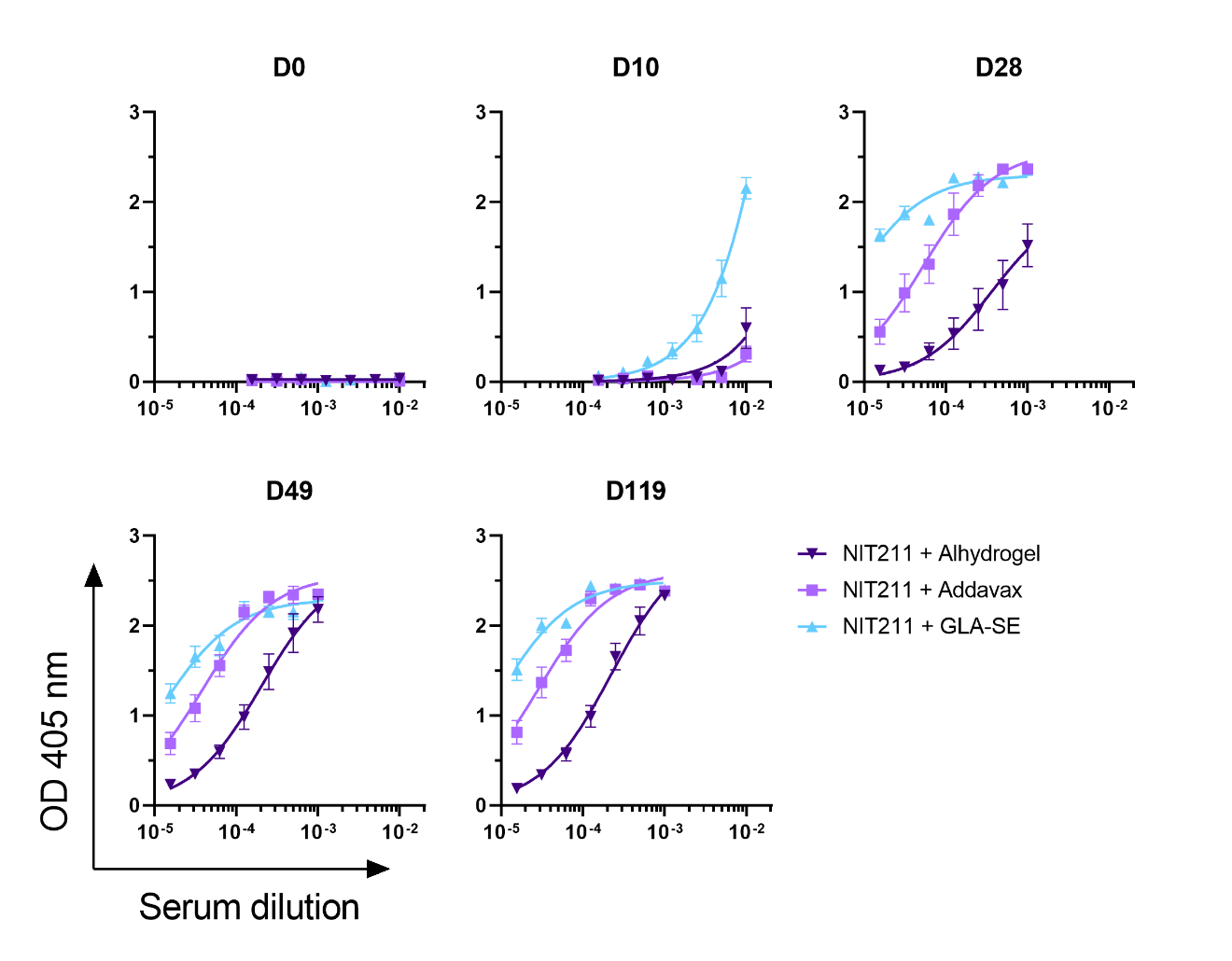


**Supplementary Figure 3. NIT211 formulated in Alhydrogel, AddaVax or GLA-SE elicits robust IgG response to CRM_197_.** Trianni mice (n = 5/group) were immunized subcutaneously on days 0, 21, 42 and 105 with NIT211 formulated in Alhydrogel, Addavax or GLA-SE and sera collected on days 0 10, 28, 49 and 119. Shown is total IgG binding to CRM_197_. Graphs depict mean values for the five serum samples from each animal group, with error bars denoting the standard error of the mean.


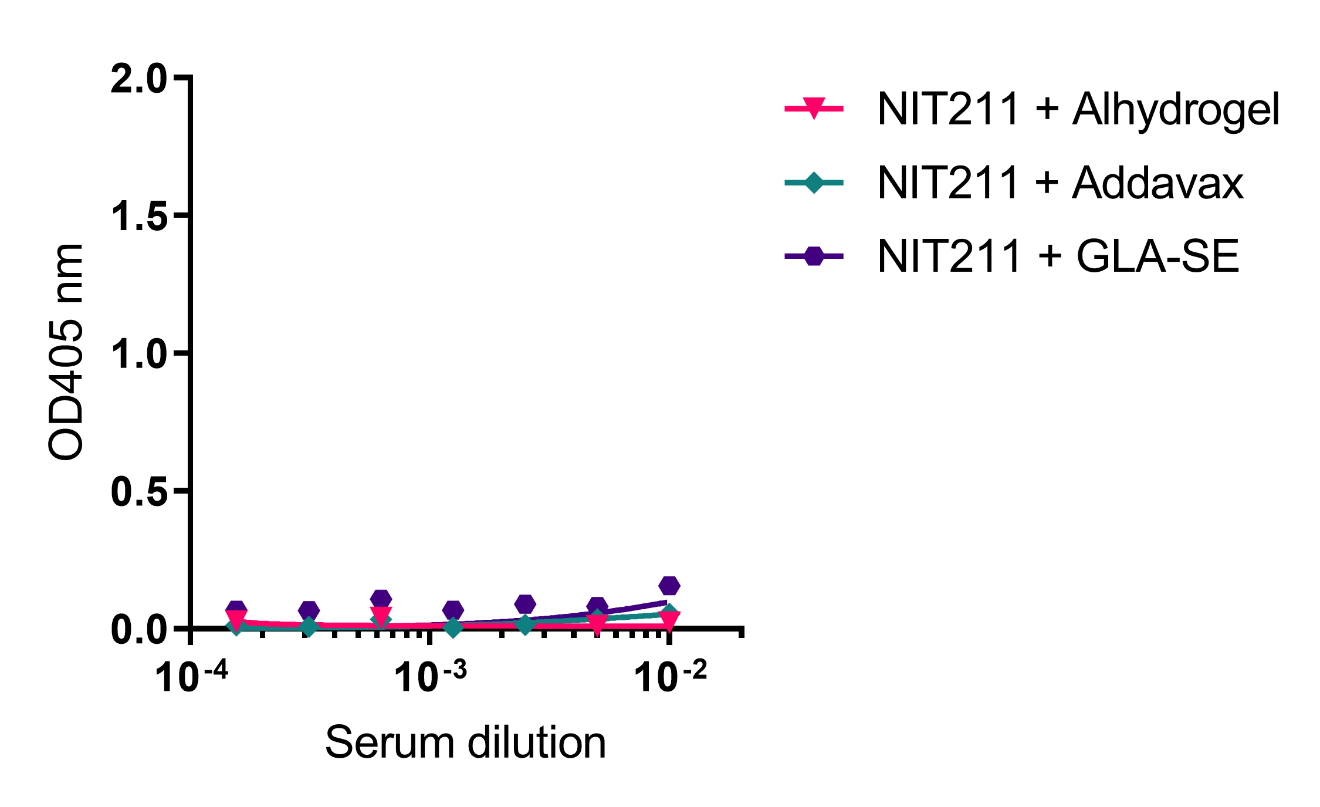


**Supplementary Figure 4. Confirmation that Trianni mouse sera collected post-immunization do not bind BSA.** Sera collected on day 119 from animals immunized with N211 formulated in Alhydrogel, Addavax and GLA-SE, respectively, were assayed for binding to BSA by ELISA to assess the level of potential non-specific binding. Graphs depict mean values for the five serum samples from each animal group. Error bars represent the standard error of the mean.


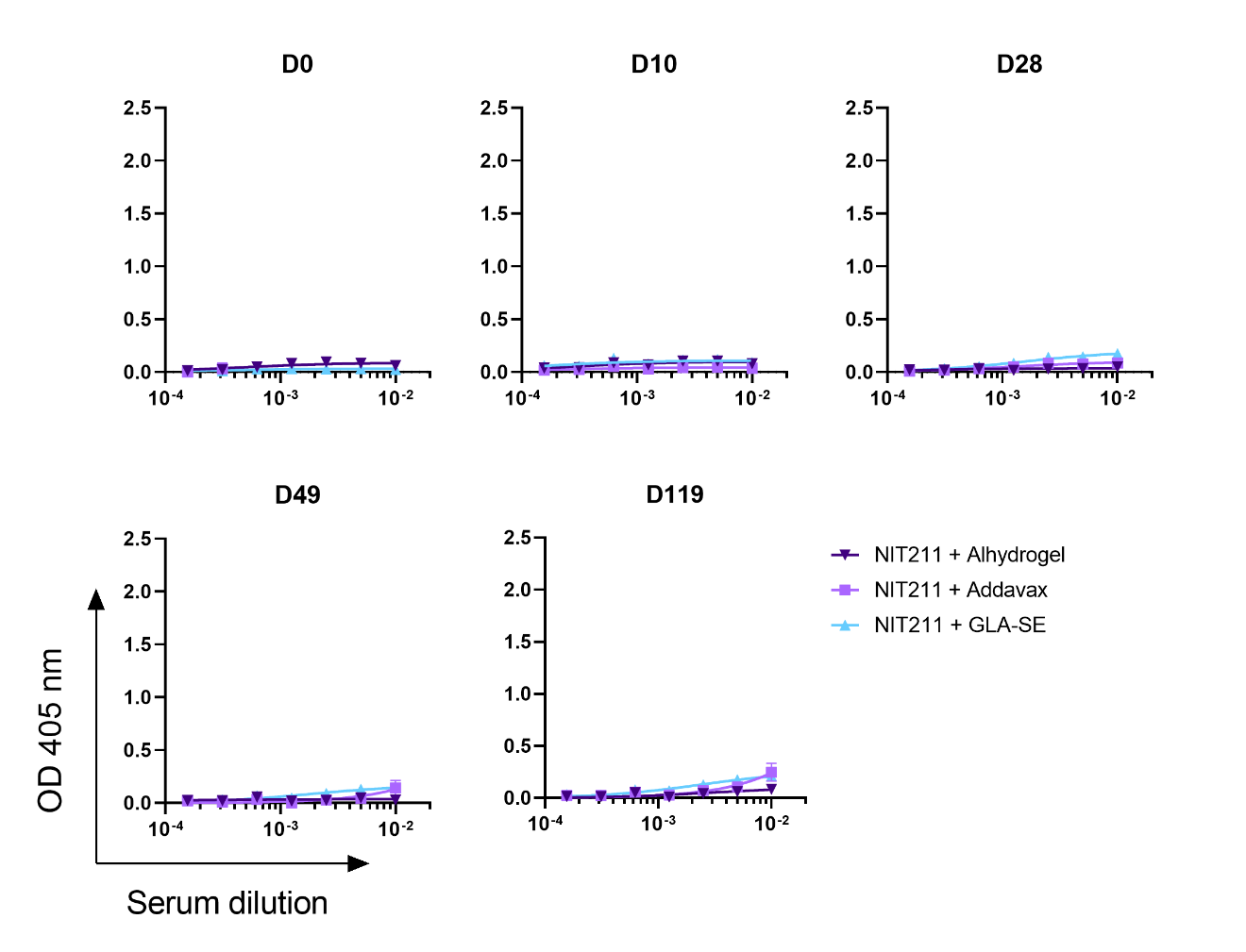


**Supplementary Figure 5. NIT211 formulated in Alhydrogel, AddaVax or GLA-SE does not evoke substantial IgM responses to the oligomannose mimetic.** The IgM response to the oligomannose mimetic was assessed by ELISA with the heterologous BSA conjugate NIT82b. Binding of total IgG antibodies in pre-immune and post-immune sera to the oligomannose mimetic BSA-conjugate. Graphs depict mean values for the five serum samples from each group.


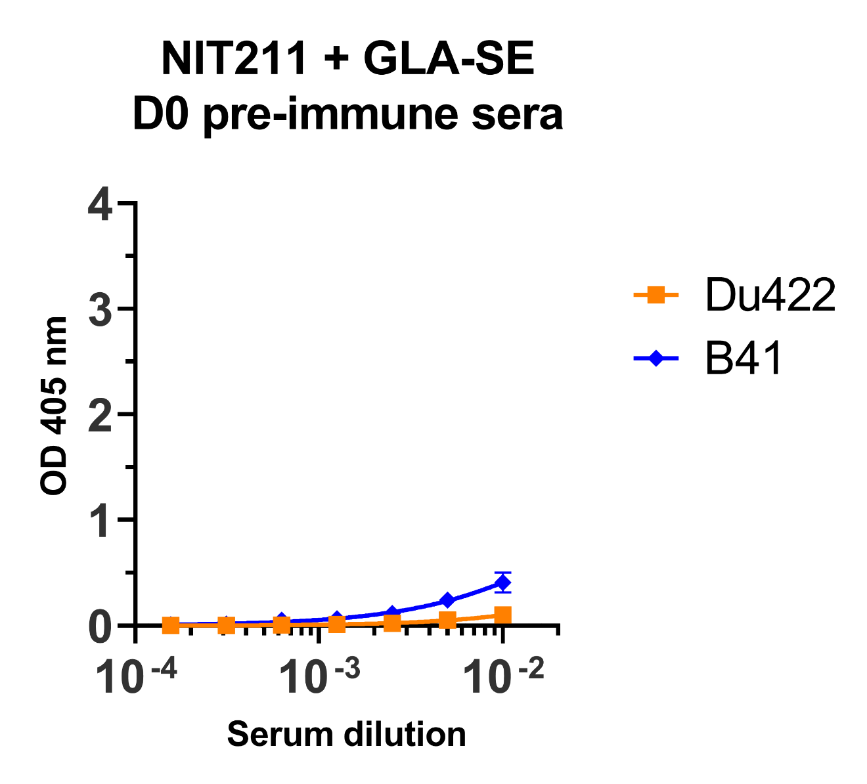


**Supplementary Figure 6. Pre-immune sera from Trianni mice immunized with GLA-SE-formulated NIT211 do not bind HIV SOSIP trimers.** Pre-immune sera collected just prior to immunization on day 0 from the NIT211 + GLA-SE group (n=5 animals) were assayed for binding to Du422 and B41 SOSIP trimer by ELISA. Graphs depict mean values for the five serum samples from each animal group. Error bars represent the standard error of the mean.


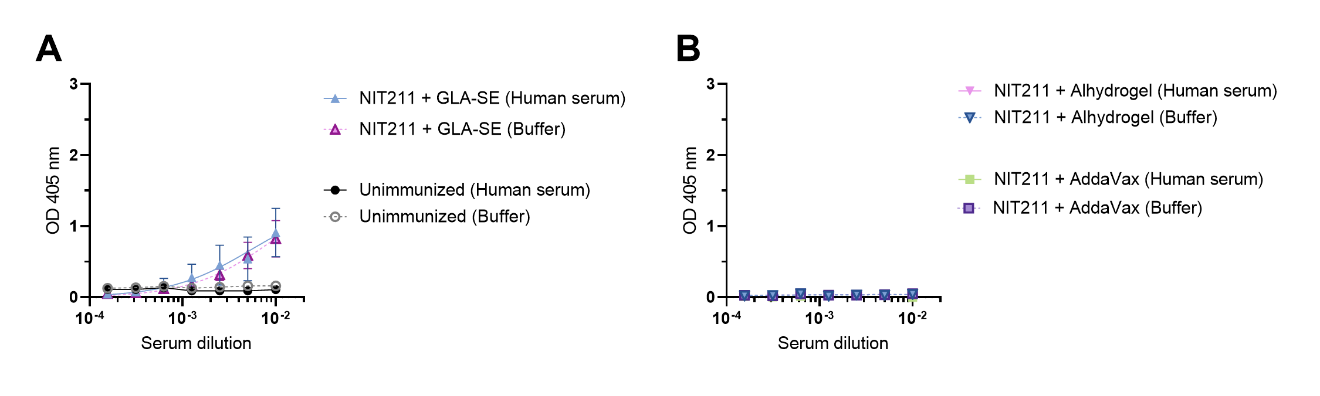


**Supplementary Figure 7.** **Sera collected at the end of a serial immunization of Trianni mice inoculated with adjuvanted NIT211 do not bind mannosidase-trimmed oligomannose mimetic preferentially.** Sera collected on day 119 were assayed for binding to the BSA-conjugated oligomannose mimetic after overnight incubation of glycoconjugate-coated ELISA plate wells with untreated human serum (Sigma) to achieve mannosidase trimming *in situ*^20^ or with buffer control. (A) Binding of sera collected on day 119 (i.e., two weeks after the last booster injection) from animals immunized with NIT211 adjuvanted with GLA-SE and of sera from unimmunized Trianni mice collected at the same timepoint (B) Binding of day 119 post-immunization sera from animals immunized with NIT211 adjuvanted with Alhydrogel and AddaVax, respectively. All binding curves depict geometric mean values for the five serum samples from each immunization group, each assayed in duplicate, with error bars denoting the standard error from the mean.

| **Supplementary Table 1. Assessment of neutralizing activity in sera from unimmunized Trianni mice and Trianni mice immunized with NIT211 glycoconjugate formulated in adjuvant GLA-SE .** | | | | | | | | | |
| --- | --- | --- | --- | --- | --- | --- | --- | --- | --- |
|  |  | **Virus Panel** | | | | | | | |
|  |  | **92TH021** | **92RW020** | **94UG103** | **92BR020** | **97ZA012** | **JRCSF** | **NL43** | **VSV** |
| **TRN_NIT211-GLA-1** | **ID_50_ (1/dil'n)** | 103 | 67 | 80 | 75 | 69 | 89 | 149 | 53 |
| **TRN_NIT211-GLA-2** |  | 49 | 40 | 52 | 43 | 46 | 71 | 97 | 43 |
| **TRN_NIT211-GLA-3** |  | <30 | <30 | <30 | 31 | 45 | 66 | 77 | 46 |
| **TRN_NIT211-GLA-4** |  | 40 | 31 | 33 | 41 | 55 | 61 | 91 | 32 |
| **TRN_NIT211-GLA-5** |  | 43 | 39 | 37 | 39 | 50 | 62 | 92 | 31 |
| **TRN_UI-1** |  | 39 | 34 | 37 | 38 | 58 | 62 | 63 | 32 |
| **TRN_UI-2** |  | 87 | 69 | 65 | 69 | 113 | 122 | 128 | 56 |
| **TRN_UI-3** |  | 90 | 40 | 48 | 59 | 92 | 70 | 176 | 37 |
| **PGT128_gl_human_IgG1** | **IC_50_ (ug/mL)** | >50 | >50 | >50 | >50 | >50 | >50 | >50 | >50 |
| **PGT128_mat_human_IgG1** |  | **<0.0977*** | **<0.0977*** | **<0.0977*** | **<0.0977*** | **<0.0977*** | **<0.0977*** | >50 | >50 |
| **PGT130_human_IgG1** |  | **<0.0977*** | **0.1272** | **5.9485** | **1.2455** | **4.4839** | **<0.0977*** | >50 | >50 |
| **PGT130-3L74H_human_IgG1** |  | **0.9790** | >50 | >50 | >50 | >50 | >50 | >50 | >50 |
| Mouse sera are from NIT211 + GLA-SE immunized animals at two weeks after the last booster immunization (day 119) and from unimmunized mice at the same time point. **ID50 (1/diln)** = Inhibitory Dilution of serum to inhibit virus infectivity by 50%. **IC50 (µg/ml)** = Inhibitory Concentration of purified antibody to inhibit virus infectivity by 50%. VSV, Vesicular Stomatitis Virus (used as negative control). | | | | | | | | | |
|  | | | | | | | | | |
